# Covid-19 Mortality: A Matter of Vulnerability Among Nations Facing Limited Margins of Adaptation

**DOI:** 10.3389/fpubh.2020.604339

**Published:** 2020-11-19

**Authors:** Quentin De Larochelambert, Andy Marc, Juliana Antero, Eric Le Bourg, Jean-François Toussaint

**Affiliations:** ^1^EA7329, Institute for Research in bioMedicine and Epidemiology of Sport (IRMES), Paris, France; ^2^Centre de Recherche sur la Cognition Animale (CRCA), Centre de Biologie Intégrative (CBI Toulouse), Université de Toulouse, CNRS, UPS, Toulouse, France; ^3^CIMS, Hôtel-Dieu, Assistance Publique—Hôpitaux de Paris, Paris, France

**Keywords:** COVID-19, demography, environment, public health, lockdown, niche adaptation

## Abstract

**Context:** The human development territories have been severely constrained under the Covid-19 pandemic. A common dynamics has been observed, but its propagation has not been homogeneous over each continent. We aimed at characterizing the non-viral parameters that were most associated with death rate.

**Methods:** We tested major indices from five domains (demography, public health, economy, politics, environment) and their potential associations with Covid-19 mortality during the first 8 months of 2020, through a Principal Component Analysis and a correlation matrix with a Pearson correlation test. Data of all countries, or states in federal countries, showing at least 10 fatality cases, were retrieved from official public sites. For countries that have not yet finished the first epidemic phase, a prospective model has been computed to provide options of death rates evolution.

**Results:** Higher Covid death rates are observed in the [25/65°] latitude and in the [−35/−125°] longitude ranges. The national criteria most associated with death rate are life expectancy and its slowdown, public health context (metabolic and non-communicable diseases (NCD) burden vs. infectious diseases prevalence), economy (growth national product, financial support), and environment (temperature, ultra-violet index). Stringency of the measures settled to fight pandemia, including lockdown, did not appear to be linked with death rate.

**Conclusion:** Countries that already experienced a stagnation or regression of life expectancy, with high income and NCD rates, had the highest price to pay. This burden was not alleviated by more stringent public decisions. Inherent factors have predetermined the Covid-19 mortality: understanding them may improve prevention strategies by increasing population resilience through better physical fitness and immunity.

## Introduction

Out of the many environmental options, human populations have concentrated in the most favorable development niche, characterized by a local mean annual temperature around 11–15°C (1), corresponding to a narrow latitude strip. In the plains of that strip, the highest life expectancies have been experienced by the populations and most of the human longevity maxima have been recorded (2), showing that the niche coincides with and allows for the highest capacities of the human physiological development (1) and wealth creation, associated with elevated gross domestic product (GDP) (3, 4).

Experiencing a recent phase of stagnation, nations encounter intrinsic and extrinsic limits: plateauing has been demonstrated in the progression of life expectancy (5–7), adult height (8), or physiological maxima (9, 10). As a consequence, societies seem to face reduced margins of adaptability (2, 10, and become more susceptible to new constraints. In fact, individuals have a limited organism shaped by physical (11) and evolutionary constraints (12), and modulated by their interactions with the environment, resulting in an age-related decline in performances (10) with a potential maximal longevity (7). Hence, global threats may put the human development niche at higher risks. Demographical, social, economic, and health parameters may underline population vulnerabilities following the recent development phase.

Countries with the highest life expectancy have demographically transitioned to greater proportions of older and frailer populations, susceptible to increased mortality rates when facing physical or biological aggressors, such as temperature elevations (13) or infections (14). Concomitantly, the causes of death in these nations have transitioned from infectious to chronic diseases: mainly cardio-vascular diseases (CVD), metabolic (diabetes, high blood pressure), and neuro-degenerative diseases or cancers. In addition, metabolic and CVD risk factors associated with high death rates, such as sedentary lifestyle, poor nutrition quality, or obesity, have a large prevalence in high income countries (15, 16) and rise in developing ones (17–19). Such comorbidities were early associated with a higher risk of death from Covid-19 (20).

The balance between the prior demographic, environmental, economic, health, or social factors in each nation may partially determine Covid-19 mortality rates, as well as the efforts made by governments to contain the pandemic. We hypothesized that nations characterized by limited progression of life expectancies, with high chronic disease rates, metabolic comorbidities, and high GDP produced higher vulnerabilities to Covid-19 and were associated with higher mortality rates during the first 8 months phase of the pandemic.

Hence, this study aimed to investigate the power of associations between Covid-19 death rates and demographic (e.g., life expectancy and its progression), health (e.g., major risk factors and lifestyles), environmental (temperature, humidity), and economic parameters (e.g., GDP and development index) as well as indices characterizing the governments' responses (e.g., stringency and containment measures) in every country affected by the pandemic.

## Methods

### Studied Countries

From the 188 countries that have declared at least one case, only those counting a minimum of 10 deaths due to Covid-19 up to the study end point (31th August 2020) were included. China and US were also analyzed by states or regions, when each of them reached the 10 deaths threshold.

### Variables of Interest

The studied outcome was the death rate due to Covid-19. Its association was tested with environmental [temperature, humidity, ultra-violet (UV) index]; demographic [life expectancy (LE), progression of LE]; health (CVD death rate, cancer death rate, infectious diseases death rate, obesity rate, sedentary, or inactive lifestyle); GDP and with each government response (containment and health index, stringency index, and economic support index).

The mortality rate due to Covid-19 was calculated as the ratio between the total number of deaths and the population size of each country, state, or region. It can be displayed as the number of deaths per 100,000 inhabitants and/or transformed into its decimal logarithm.

To test the optimal development niche effect, the Covid-19 mortality rates were analyzed according to the latitude and longitude of each country. Both were characterized by the barycenter of the country (GPS coordinates). Likewise, each state in the USA and each region in China was analyzed with its own latitude and longitude [as reported on the Center for Systems Science and Engineering (CSSE) at Johns Hopkins University (JHU)] for the environmental analysis.

### Data Collection

Daily data on the number of cases and deaths due to Covid-19 were collected up to the study end point via the Johns Hopkins University data source (https://github.com/CSSEGISandData/COVID-19). The latest population sizes available, used to calculate the mortality rate were extracted from the UNdata website (http://data.un.org/Data.aspx?d=POP&f=tableCode%3A22). The same data source was used to obtain the GDP for each country, the last year with available data being used.

Daily environmental data (temperature, humidity, and UV index) were collected via the Darsky website (https://darksky.net/). They were recorded from the beginning of the pandemic (defined as the day when the country reached a total of 10 deaths due to Covid-19) until the peak of the pandemic. To calculate the pandemic peak (PP), the number of cumulative deaths was theorized with a non-symmetrical logistic regression:

$$\begin{array}{l} Y\left(t\right)=c+\;\frac{d-c}{{\left(1+\exp\left(b\left(\log\left(t\right)-\log\left(e\right)\right)\right)\right)}^{f}} \end{array}$$

where *Y*: logarithm of the number of deaths per 100,000 population

$$\begin{array}{r} t\;:\text{ time in days}\\ b,c,d,e,f\;:\text{ model parameters} \end{array}$$

The parameter *d* controls the height of the asymptote of the curve. The parameters *b* and *f* jointly control the magnitude, which represents the speed of transition between the two asymptotes. The parameter *e* controls the position of the slope and the parameter *c* the left asymptote of the curve.

The maximum of the derivative of this function was used to determine PP in each country. We calculated the mean of each of the three variables (temperature, humidity, and UV) in each country for a period starting at the beginning of the epidemic and ending the day of the PP.

Several countries (e.g., India, Argentina, etc.) have not yet reached the peak of the epidemic first wave. In order to take this parameter into account, the analyzes were also done with a death number that was estimated at the 99% time point (i.e., when the epidemic reaches 99% of the total death toll from the first epidemic wave) according to the logistic regression above. The estimated number of deaths for each country as well as all analyzes with such simulated data are presented in the Supplementary Material (Supplementary Table 1, Supplementary Figures 1–3), where the actual number of deaths for each country at the last known date is compared to the theoretical number according to the model.

The geographical coordinates corresponding to the barycenter of each country were retrieved thanks to the package *rgeos* in the R software. Latitude is expressed negatively in the southern hemisphere, positively in the northern one. Relative to the Greenwich meridian, longitude is expressed negatively for the western countries, positively for the eastern ones.

The obesity rate was calculated as the percent of the country total population considered to be obese, according to the last year this prevalence data was publicly available. Adult obesity is defined through a Body Mass Index (BMI) greater to or equal to 30 kg/m^2^. Data were collected from *The World Factbook* from the US intelligence agency (https://www.cia.gov/library/publications/the-world-factbook/fields/367.html).

The inactive lifestyle was characterized by the prevalence (percentage of the population) of adults performing <150 min of moderate-intensity physical activity per week, or <75 min of vigorous-intensity physical activity per week, or equivalent. Data were retrieved from the website of the World Health Organization (https://apps.who.int/gho/data/node.main.A893?lang=en). This prevalence is based on self-reported physical activity captured using the GPAQ (Global Physical Activity Questionnaire), the IPAQ (International Physical Activity Questionnaire), or a similar questionnaire covering activity at work/in the household, for transport, and during leisure time.

The current life expectancies were collected from the World Bank, based on the last year these data were available (https://data.worldbank.org/indicator/SP.DYN.LE00.IN). To calculate the progression of LE, we used data from 2010 up to now. The α coefficient of the linear regression between current LE and the 2010 one was determined to estimate the progression trend. The greater the index, the greater the life expectancy gains in the last decade.

The burden resulting from major chronic diseases (CVD, metabolic diseases, cancer) and from infectious diseases in the previous population death rates was estimated by the proportion of the mortality rates associated with these major causes compared to the all-cause mortality rates. Both sexes and all age classes were taken into account. Data were retrieved from the IHME “GDP results tools” (http://ghdx.healthdata.org/gbd-results-tool) up to the last year the mortality rate was available. They appear as “Neoplasms death rate,” “CV and MD death rate,” and “Infectious diseases death rate.”

We used the Oxford university data source to characterize the state responses, regarding the containment and health index, the stringency index, and the economic support index (https://www.bsg.ox.ac.uk/research/research-projects/coronavirus-government-response-tracker) including public health measures taken by each country at short term. The Oxford COVID-19 Government Response Tracker (OxCGRT) systematically collects information on several different common policy responses that governments have taken to respond to the pandemic on 17 indicators. The data from the 17 indicators were aggregated into a set of three common indices, reporting a number between 1 and 100 to reflect the level of government's action on each topic: (1) the containment and health index combines lockdown restrictions and closures with measures such as testing policies and contact tracing, short term investment in healthcare, as well as investments in vaccines; (2) the economic support index records measures such as income support and debt relief; (3) the original stringency index records the strictness of lockdown and policies that primarily aimed at restricting population mobility.

### Statistical Analysis

To study the relationship between environmental variables and the Covid-19 mortality rate, we carried out a linear (*y* = α.*x*+β) and a two-degree polynomial $(y=\alpha_{1}.x+\;\alpha_{2}.x^{2}+\beta$) analysis, taking into account the notion of physiological optima (21) through an optimized link between thermodynamics and physiology/pathology (parameters of air-borne diseases such as influenza also show a maximal transmission rate for a specific range of ambient temperatures–20). For each of the three environmental variables, we kept the best of the two models based on the adjusted coefficient of determination, taking into account the complexity of the model.

To test potential associations between the studied parameters, a Principal Component Analysis (PCA) was computed. Pearson correlation coefficients and tests for association were computed to measure the correlation between each pair of parameters. The results are presented in a correlation matrix. For these analyzes, we used the absolute value of latitude, representing the deviation from latitude 0. Finally, when a polynomial regression was determined for environmental variables, the deviation from the maximal value was used to test the association with Covid mortality.

Results are considered significant at *p* < 0.05. All statistical analyses were performed with R (version 3.6.1; The R Foundation for Statistical Computing, Vienna, Austria).

## Results

One hundred and sixty countries were included in the study (Supplementary Table 1), accounting for a total of 846,395 deaths due to Covid-19 up to the study end point (31th August 2020).

### Covid-19 Mortality and the Global Niche

**Figure 1:**
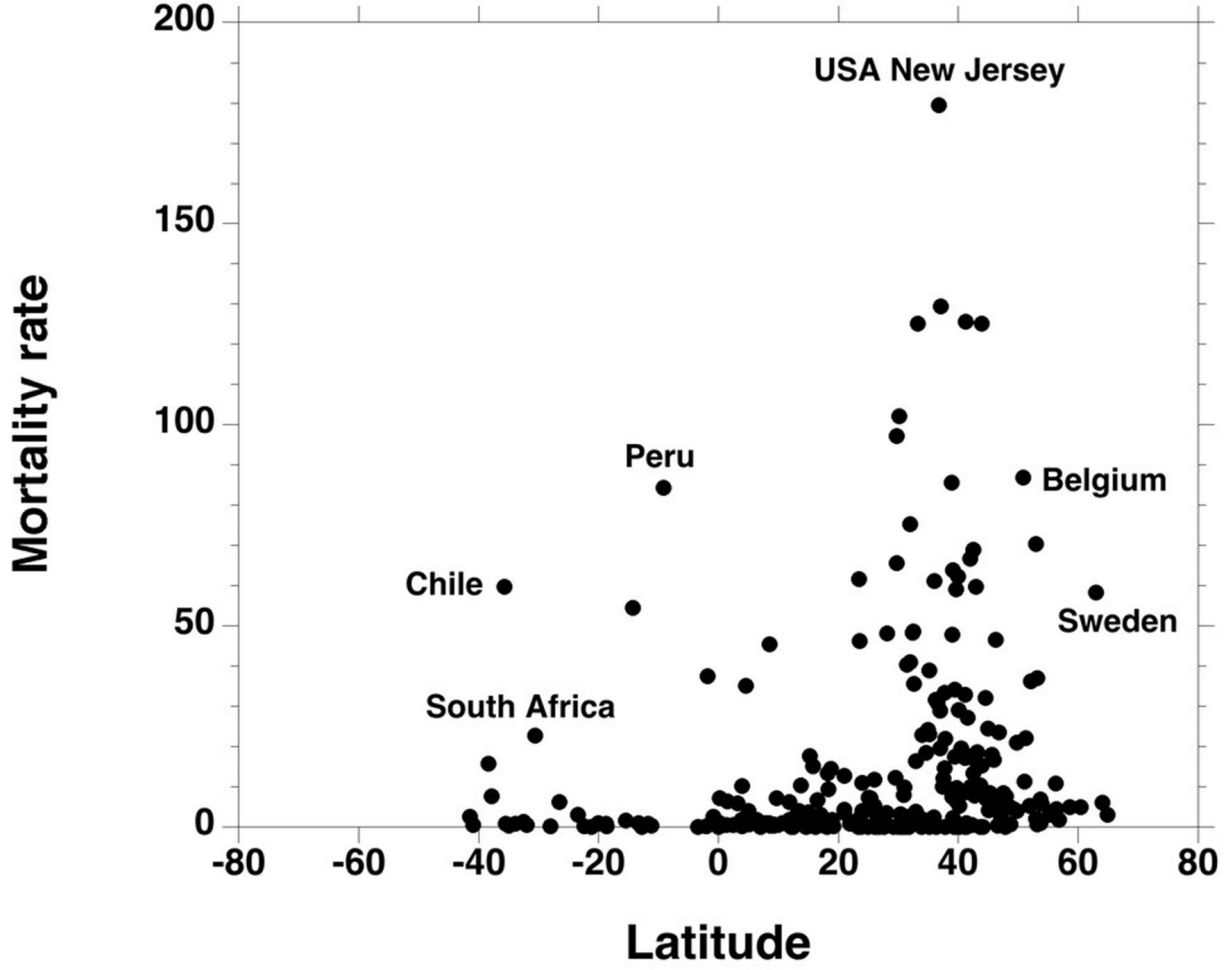
Each point represents the Covid-19 mortality rate of a country or a state, according to its latitude. Greater mortality rates were mostly observed in the [25 /65] latitude interval.

The geographical relation between Covid-19 mortality rate and latitude shows that higher mortality rates were observed in the 25/65° northern parallels (Figure 1, Supplementary Figure 4). The [25–65°] latitude intervals (North and South) delimited an area where 78% of all Covid-19 deaths were recorded (in the European continent, this area includes Spain and Italy up to the southern part of Sweden; in the Americas, it covers the state of Texas up to the Hudson Bay; the southern part of Brazil and the states under it; in the African continent: the Maghreb states and South Africa). This area includes the states with the highest recorded death rates (New-Jersey in the Americas, Belgium in Europe).

**Figure 2:**
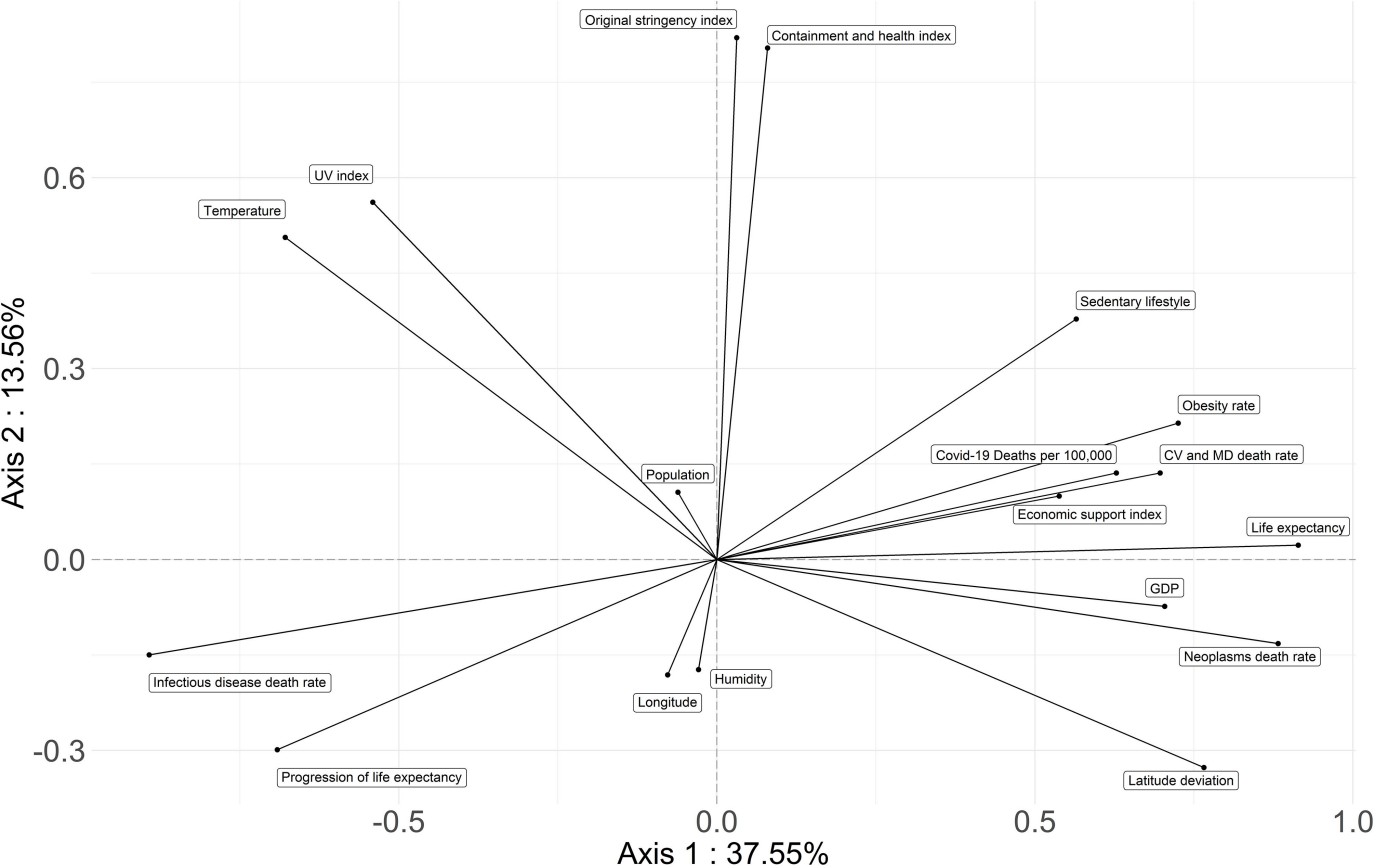
PCA first factorial plane: axis 1 is horizontal; axis 2 is vertical. The more distant a variable is from the center, the more it is correlated with the first or the second factorial axis. The studied parameters regrouped the countries associated with Covid-19 highest death rates on the right of the horizontal axis. These are the high income countries with a high LE but a low progression of it, high sedentarity, obesity, high deviation from latitude 0 and low longitude (Asian countries have a high longitude, while it is negative in the Americas). Countries associated with low Covid-19 death rates have a low GDP, a low LE but a great margin of progression for it, a high prevalence of infectious diseases, a greater deviation from optimum temperature and UV index; they occupy the left part of the axis. Lockdown stringency, containment index and ambiant humidity are not correlated with Covid-19 mortality, as they are linked to the second axis. The cloud of individuals on the first factorial plane is presented in the Supplementary Figure 5.

Negative longitudes (American Countries) were also associated with higher death rates (Figure 2). The [−35/−125°] longitude interval (West) delimited an area where 57% of all Covid-19 deaths were recorded.

### Covid-19 Mortality and the Environment

**Figure 3:**
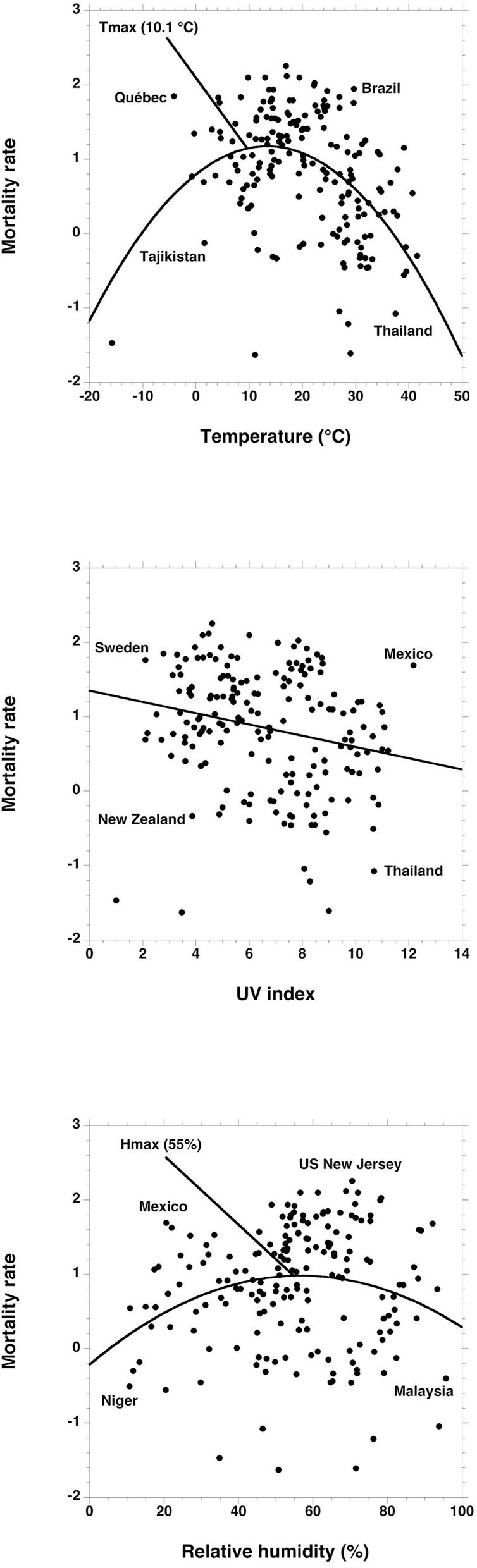
Polynomial or linear regression between the logarithm of the mortality rates due to Covid-19 and the UV index, humidity, and average temperature from the local beginning of the pandemic up to the peak.

Polynomial regression was used for the relationship between the number of deaths per 100,000 inhabitants according to temperature (*R*^2^ = 0.21) and humidity (*R*^2^ = 0.05) (Figure 3). A linear relationship was preferred for the UV index (*R*^2^ = 0.11). Maximal death rates are obtained for a temperature *T*_max_ of 10.1°C, a humidity *H*_max_ of 55%, and a zero UV index. Deviations from *T*_max_ and *H*_max_ were used for the multifactorial analysis of death rates with temperature and humidity.

### Principal Component Analysis

Combining the studied parameters, the first and second factorial planes of the PCA represent 60.27% of the information (Figure 2). The first axis concentrates 37.55% of total inertia and axis 2 represents 13.56% of it. The third factorial axis represents 9.16% of the information. The cloud of individuals on the first factorial plane is presented in Supplementary Figure 5.

**Figure 4:**
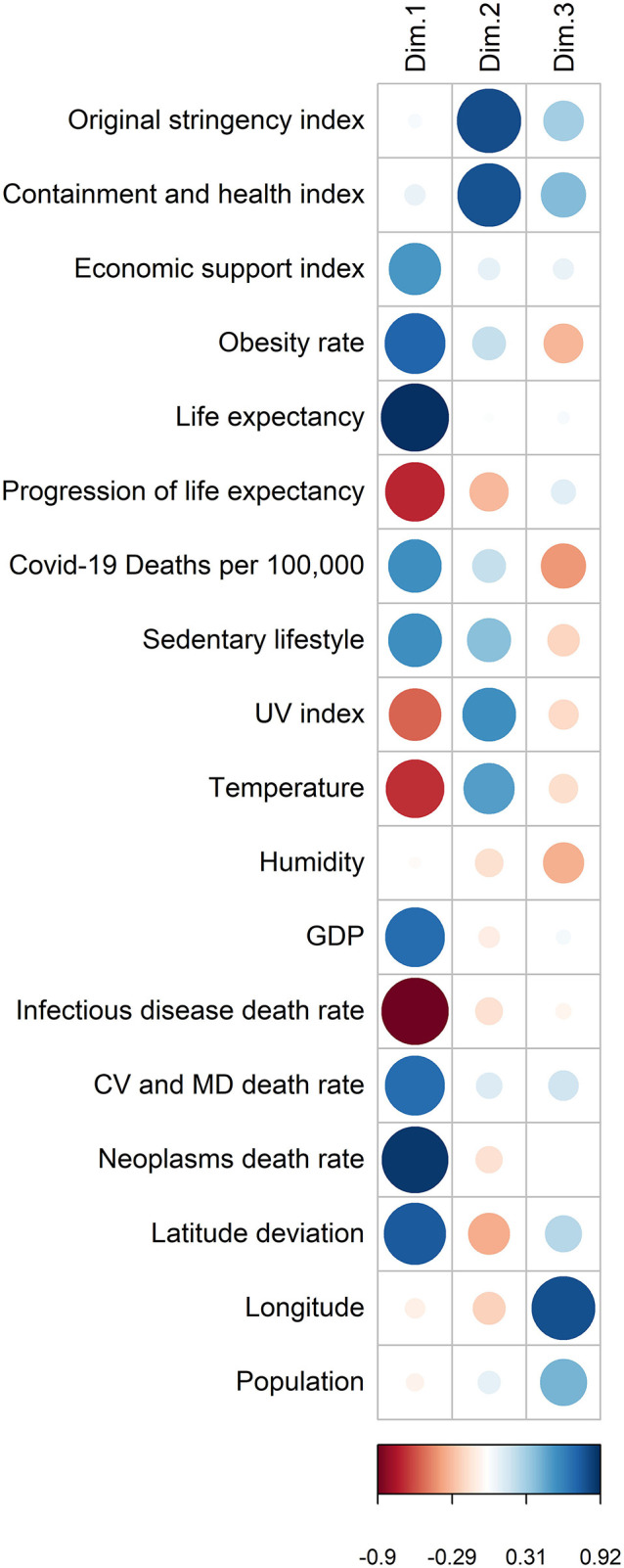
Coordinates of the variables on the first 3 factorial axes. The larger the circle, the more the variable is correlated with the axis. A blue circle indicates that the variable is negatively correlated, a red circle indicates that the variable is positively correlated. The scale shows the coordinates of the variables on the axis.

**Figure 5:**
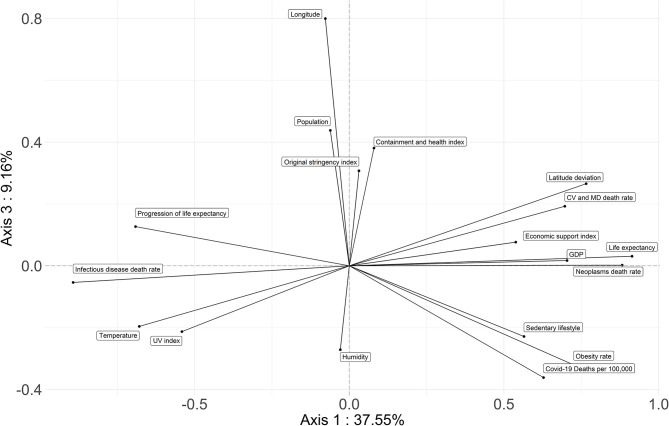
PCA third factorial plane: axis 1 is horizontal; axis 3 is vertical. The studied parameters regrouped the countries associated with Covid-19 highest death rates on the right of the horizontal axis. Longitude and obesity rates are related to the third axis.

The first axis of PCA opposes two groups of countries (Figures 2, 4, 5). High income northern countries are positively correlated to this axis: they provide high economic support, have higher LE but lower progression of LE, more frequent sedentary lifestyle, larger obesity rates, and higher mortality from CVD and cancer. Occupying the left part of the axis are countries with a low GDP, lower life expectancy but greater progression of LE, higher death rate from infectious diseases, greater deviation from optimum temperature, and UV index. Covid-19 death rate is higher in countries strongly and positively correlated with the first factorial axis on the right.

The government's responses (i.e., the severity index and the containment and health index) are strongly correlated with the second factorial axis (Figures 2, 4). The death rate from Covid-19 is not correlated with this axis. Therefore, the death rate appears not to be linked with the responses of governments.

The third axis shows a relationship between Covid-19 mortality and longitude as well as obesity and sedentarity (Figures 4, 5). American countries have a higher obesity rate and a higher Covid-19 mortality rate; Asian countries have lower obesity rates and lower Covid-19 mortality rates.

**Figure 6:**
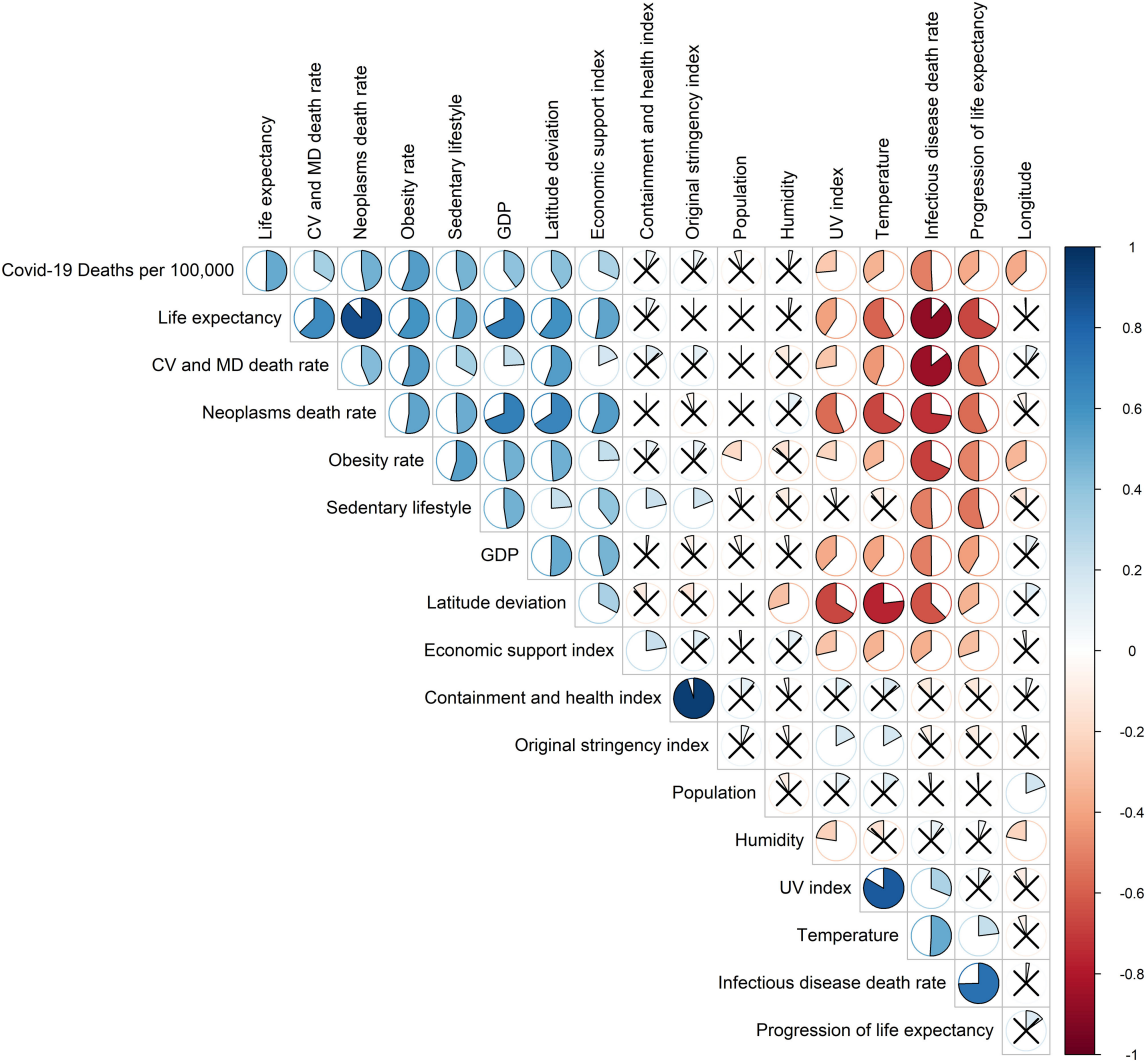
Correlation matrix: a larger area in the circles indicates a stronger correlation between the row and the column variables. A blue circle indicates a positive correlation coefficient; a red circle indicates a negative one; a full circle corresponds to *r* = 1 or −1; an empty circle corresponds to *r* = 0. If the Pearson correlation test was not significant, a cross on the circle was added.

The correlation matrix (Figure 6) shows that the Covid-19 mortality rate is positively correlated to a group of variables composed of the inactive lifestyle (*r* = 0.46, *p* < 10^−6^), obesity rate (*r* = 0.55, *p* < 10^−11^), GDP (*r* = 0.40, *p* < 10^−7^), economic support index (*r* = 0.31, *p* < 10^−3^), life expectancy (*r* = 0.50, *p* < 10^−11^), burden of mortality due to CVD (*r* = 0.33, *p* < 10^−3^), cancer (*r* = 0.47, *p* < 10^−9^), and deviation from latitude 0 (*r* = 0.41, *p* < 10^−3^). The mortality rate due to Covid-19 is negatively correlated to another group of variables composed of the mortality rate from infectious diseases (*r* = −0.50, *p* < 10^−9^), the progression of life expectancy (*r* = −0.37, *p* < 10^−4^), longitude (*r* = −0.36, *p* < 10^−3^), the deviation from optimum temperature (*r* = −0.39, *p* < 10^−5^), UV index (*r* = −0.37, *p* < 10^−43^). There is no significant correlation with the deviation from optimum humidity (*r* = 0.03, *p* = 0.52), the containment and health index (*r* = 0.07, *p* = 0.51), the original stringency index (*r* = 0.07, *p* = 0.36), and population size (*r* = −0.05, *p* = 0.35). A negative correlation also relates obesity and longitude (*r* = −0.33, *p* < 10^−4^).

The principal component analysis as well as the correlation matrix with the estimated data are presented in Supplementary Figures 1–3. The analyzes with the estimated death number at the end of the first epidemic wave do not change the conclusions of the analyzes on the real data. The direction of the correlations as well as their significance in the correlation matrix are unchanged as well.

## Discussion

### Main Findings

This analysis shows that higher Covid-19 mortality rates are mostly found in countries experiencing higher life expectancies and showing a recent slowdown of this progression. Most of these developed and aging societies are latitudinally located over the 25° parallel. They also have higher GDP and chronic diseases levels (e.g., CVD and cancer) associated with major metabolic risk factors (e.g., inactive lifestyle, sedentarity, and obesity). High temperature and UV levels are associated with low death rates such that northern and western countries pay the most severe toll to Covid-19.

In the PCA, the first axis shows a strong correlation between Covid-19 death rates and countries inside the [25/65°] latitudinal strip, while the third axis reveals two correlations with Covid-19 death rates: one with longitude, a second one with obesity. This suggests that states in the Americas plagued with frequent inactive lifestyle and higher obesity rates than Asian countries experienced a higher death toll.

This is consistent with the hypothesis of an optimal human development niche, that has aggregated favorable health, demographic, environment, and economic parameters (1). However, though previously positive, they now expose populations to higher vulnerabilities to both infectious (Covid-19) or physical constraints (heat waves). Regarding government's actions (i.e., containment and stringency index), no association was found with the outcome, suggesting that the other studied factors were more important in the Covid-19 mortality than political measures implemented to fight the virus, except for the economic support index. It may however be important to decipher this positive relation in a plausible chronological order: it does not seem that a higher economic support would induce a higher Covid-19 mortality, but rather that a higher death toll rate provoked a larger societal reaction, including a higher amount of economic measures, when available.

The design of this study aimed to draw a global description of the Covid-19 mortality and its associations with several major parameters. It is out of scope to speculate on any cause-effect relation. Nevertheless, some explanatory hypothesis may be proposed: countries displaying greater susceptibility (determined by a more fragile balance between health, demographic, environment, and economic parameters) seem to have narrower margins of adaptation and be therefore more vulnerable to main aggressors.

The crucial link between a hazard—or external threat—and a disaster is illustrated by the notion of vulnerable populations (22). Vulnerability is the result of complex interactions of distinct risks, exposure to the threat, and the lack of defenses or resources to deal with it. During a pandemic situation, the foremost indicator of countries health fragility may be seen in the proportion of older people (who were the SARS-CoV-2 major target), given the ineluctable diminished performances and resilience with age (23). Resulting from both biological and social processes, the decline in health and physical strength and the increasing disabilities particularly affect old people, bringing them closer to vulnerability thresholds. The highest proportions of elderly people are observed in countries with higher life expectancy (24, 25). Such nations may suffer from higher mortality levels when new aggressors appear.

Previous studies have illustrated the relation between frailty and mortality (26). For instance, the 2003 heatwave killed 30,000 to 50,000 people in Europe and 15,000 in France (13, 27), 80% of them being elderly people. Among centenarians, who are more likely to decline suddenly, mortality due to infections increases (e.g., pneumonia) (14). Accordingly, the Covid-19 mortality was the highest among the elderly worldwide (28). Moving toward higher life expectancy will therefore expose greater proportions of people to high mortality rates, especially when facing mass threats or when environment conditions largely evolves.

Concomitantly to a high life expectancy, the development afforded by an elevated GDP usually favors inactive lifestyles, sedentary behaviors, and obesity (15, 29), increasing the risk for hypertension, diabetes, and CVD, the most frequent comorbidities associated with Covid-19 mortality (30–32). With an epidemiological transition toward more prevalent chronic diseases, countries with high life expectancy have also increased concurrent risks, restraining their adaptability margins.

The associations found among two opposed groups of countries suggest important inherent factors, predetermining the consequences of global threats. Properly understanding the relations between those parameters may help to provide new prevention strategies. Covid-19 has prompted a wide range of responses from governments around the world, yet the contagion and mortality curves are very homologous among countries (33). This is reinforced by our findings regarding the lack of any association with the government's actions taken during the pandemic. In that sense, the determining demographic, health, development, and environment factors seem much more important to anticipate the lethal consequences of the Covid-19 than government's actions, especially when such actions are led by political goals more than by sanitary ones. This last result however cannot predict that other types of measure would not reduce the pandemia death load.

This study highlights the great difficulties of adaptation that most countries will face (34, 35). Climate change for instance will disturb the optimal niche by forcing the ideal development temperature toward north. Infection balance and human resilience supported by local species equilibrium may be impaired as a result. Understanding where the risks and weaknesses are in each country is an important starting point when preparing to face new threats. In the Covid-19 case, an advisable strategy may be to increase populations immunity and resilience (36) and prevent sedentary behaviors through higher physical activity and better physical fitness. Hence, political strategies restricting physical activity (e.g., closing sport facilities) may refrain the enhancement of population immunity in response to present and future viral aggressors.

The first limitation of this study is the uncertainty and reliability of the recorded national data on Covid-19 deaths, given the diverse counting methods in the different countries. We also acknowledge the limit of the reliability of the input data, since it refers to worldwide data collections. However, these are the least uncertain and the most reliable sources. Furthermore, the large size of the datasets compensates for the internal variability.

Another limitation is that the pandemic is not over, with American countries displaying a kinetics partially diverging from the European ones. While a clear mortality peak was observed in Europe with a quick decrease after it, it is not the case in several American countries: Mexico, Peru, and Brazil show a lasting plateau for the time being and the USA experienced a spring peak in the eastern states and a summer peak in southern ones. If finally, Covid-linked mortality would be higher in countries of Latin America than in richer countries, it would be necessary to understand the peculiar features, absent of our analysis, explaining such a result. A large dependence to seasonal parameters may also modify some conclusions at the end of the pandemic (e.g., if mortality does not decrease for months in these countries). But it may not change the conclusions about the first phase we deal with in that study. Indeed, countries with the highest death toll could still be in the Americas as USA have already experienced a first regression of life expectancy, whereas Mexico also shows one of the highest obesity rates.

This study has focused on the explosive sub-exponential phase of Covid-19 epidemic in each country. However, a previous period of propagation has probably started in the summer or fall of 2019. It is difficult to account for it, but such a diffusion phase of SARS-CoV-2 may be investigated through both International Air Transport Association (IATA) databases and modeling of airplane transport (37). The situation in islands such as Taiwan, New Zealand, or Iceland, that quickly imposed restrictive measures on air transport, shows that the virus has not become endemic in these first 8 months. After a rapid propagation phase, only the re-importation of subjects contaminated outside the island provoked new local cases. Finally, we did not account for the various viral sub-types, that could modify the relations shown here, as they may theoretically have a different impact on death rate. The main recorded variants however did not appear to produce such a difference on mortality (38).

## Data Availability Statement

The original contributions presented in the study are included in the article/Supplementary Materials, further inquiries can be directed to the corresponding author/s.

## Author Contributions

QD, AM, JA, ELB, and J-FT conceived, designed, performed, and analyzed the research. QD and AM conceived, designed, and collected data from website. QD, ELB, and J-FT carried out the statistical analyzes. QD, JA, ELB, and J-FT wrote the manuscript. All authors read, review and approved the final manuscript.

## Conflict of Interest

The authors declare that the research was conducted in the absence of any commercial or financial relationships that could be construed as a potential conflict of interest.

## Supplementary Material

Supplementary Table 1 Number of deaths as of August 31th, 2020, number of theoretical deaths according to our model as of August 31th, 2020 and number of deaths estimated at the end of the first epidemic wave according to our model for each country included in the analysis. The estimated death number for each country was obtained from the logistic regression (see section Methods—Data collection) when the first epidemic wave was expected to reach 99% of the total death toll. The correlation matrix as well as the principal component analysis were performed with data to assess whether the results may change at the end of the first epidemic wave.

Supplementary Figure 1 First factorial plan of the principal component analysis with the estimated data (see Section Methods—Data collection). Results are the same with estimated data and actual data.

Supplementary Figure 2 Coordinates of the variables of the principal component analysis with the estimated data (see Section Methods—Data collection). Results are the same with estimated data and actual data.

Supplementary Figure 3 Correlation matrix with the estimated death numbers obtained from the logistic equation (see Section Methods—Data collection). Results are the same with estimated data and actual data.

Supplementary Figure 4 Covid-19 mortality is expressed in numbers in the top figures and in rates in the bottom figures. It is expressed in raw data on the left and in decimal logarithm on the right.

Supplementary Figure 5 The position of the countries on the graph represents their correlation according to the variables of the first factorial plane. For example, countries positively correlated to axis 1 (right), will be positively correlated to variables to the right of axis 1.
