# Supplementary material for: Covid-19 Mortality: A Matter of Vulnerability Among Nations Facing Limited Margins of Adaptation
Source: Front Public Health. 2020 Nov 19;8:604339. doi: 10.3389/fpubh.2020.604339 (PMC7710830; doi:10.3389/fpubh.2020.604339)
Supplement: Supplementary Table 1 — Number of deaths as of August 31th, 2020, number of theoretical deaths according to our model as of August 31th, 2020 and number of deaths estimated at the end of the first epidemic wave according to our model for each country included in the analysis. The estimated death number for each country was obtained from the logistic regression (see section Methods—Data collection) when the first epidemic wave was expected to reach 99% of the total death toll. The correlation matrix as well as the principal component analysis were performed with data to assess whether the results may change at the end of the first epidemic wave. [file Table_1.DOCX]

| Country name | Deaths on August 31th, 2020 | Estimated deaths at the end of the first wave (99%) | Theoretical deaths according to the model as of August 31th, 2020 |
| --- | --- | --- | --- |
| Afghanistan | 1402 | 1404 | 1393 |
| South Africa | 14028 | 14957 | 13951 |
| Albania | 280 | 289 | 273 |
| Algeria | 1501 | 2205 | 1455 |
| Germany | 9300 | 9067 | 9099 |
| Andorra | 53 | 52 | 52 |
| Angola | 107 | 112 | 106 |
| Saudi Arabia | 3870 | 4475 | 3773 |
| Argentina | 8457 | 19689 | 8671 |
| Armenia | 877 | 935 | 871 |
| Australia | 652 | 1112 | 204 |
| Austria | 733 | 703 | 705 |
| Azerbaijan | 531 | 535 | 527 |
| Bahamas | 50 | 497 | 26 |
| Bahrain | 189 | 213 | 187 |
| Bangladesh | 4248 | 5665 | 4137 |
| Belarus | 676 | 835 | 662 |
| Belgium | 9894 | 9759 | 9794 |
| Belize | 13 | 277 | 15 |
| Benin | 40 | 40 | 40 |
| Bolivia (Plurinational State of) | 4966 | 6945 | 4941 |
| Bosnia and Herzegovina | 598 | 2734 | 588 |
| Brazil | 120828 | 163656 | 119060 |
| Bulgaria | 613 | 4202 | 618 |
| Burkina Faso | 55 | 54 | 54 |
| Cabo Verde | 40 | 65 | 40 |
| Cameroon | 411 | 435 | 417 |
| Canada | 9164 | 9066 | 9078 |
| Chile | 11244 | 11925 | 11096 |
| China | 4722 | 4725 | 4742 |
| Cyprus | 20 | 19 | 19 |
| Colombia | 19363 | 26261 | 19251 |
| Congo | 78 | 164 | 78 |
| South Korea | 324 | 293 | 294 |
| Costa Rica | 418 | 533 | 414 |
| Côte D'Ivoire | 115 | 119 | 114 |
| Croatia | 184 | 172 | 158 |
| Cuba | 94 | 87 | 87 |
| Denmark | 624 | 610 | 612 |
| Djibouti | 60 | 59 | 59 |
| Egypt | 5399 | 5309 | 5269 |
| El Salvador | 713 | 867 | 711 |
| United Arab Emirates | 382 | 356 | 355 |
| Ecuador | 6555 | 6597 | 6199 |
| Spain | 29011 | 28190 | 28293 |
| Estonia | 64 | 67 | 67 |
| Eswatini | 91 | 100 | 92 |
| United States of America | 183066 | 176109 | 166611 |
| Ethiopia | 793 | 837 | 785 |
| The former Yugoslav Republic of Macedonia | 600 | 600 | 579 |
| Russian Federation | 17045 | 20414 | 16839 |
| Finland | 335 | 329 | 330 |
| France | 30611 | 29856 | 29958 |
| Gabon | 53 | 52 | 52 |
| Gambia (Islamic Republic of the) | 96 | 95 | 95 |
| Georgia | 19 | 17 | 17 |
| Ghana | 276 | 575 | 279 |
| Greece | 262 | 214 | 213 |
| Guatemala | 2740 | 3528 | 2739 |
| Guinea | 59 | 79 | 56 |
| Guinea Bissau | 34 | 34 | 32 |
| Equatorial Guinea | 83 | 100 | 88 |
| Guyana | 36 | 693 | 30 |
| Haiti | 201 | 233 | 203 |
| Honduras | 1858 | 1837 | 1765 |
| Hungary | 614 | 600 | 602 |
| India | 64469 | 240792 | 64383 |
| Indonesia | 7343 | 14428 | 7279 |
| Iran (Islamic Republic of) | 21462 | 28715 | 13264 |
| Iraq | 6959 | 8146 | 6714 |
| Ireland | 1777 | 1743 | 1749 |
| Iceland | 10 |  |  |
| Israel | 919 | 2085 | 559 |
| Italy | 35477 | 34962 | 35078 |
| Jamaica | 21 | 15 | 14 |
| Japan | 1285 | 1062 | 1063 |
| Jordan | 15 | 10 | 11 |
| Kazakhstan | 1523 | 1583 | 1527 |
| Kenya | 574 | 600 | 577 |
| Kyrgyzstan | 1058 | 1320 | 1323 |
| Kosovo | 488 | 534 | 496 |
| Kuwait | 530 | 535 | 506 |
| Lesotho | 31 | 33 | 31 |
| Latvia | 34 | 32 | 32 |
| Lebanon | 160 | 601 | 146 |
| Liberia | 82 | 82 | 82 |
| Libya | 232 | 349 | 235 |
| Lithuania | 86 | 82 | 82 |
| Luxembourg | 124 | 114 | 115 |
| Madagascar | 191 | 188 | 186 |
| Malaysia | 126 | 121 | 122 |
| Malawi | 174 | 176 | 173 |
| Maldives | 28 | 259 | 28 |
| Mali | 126 | 125 | 125 |
| Malta | 12 | 9 | 9 |
| Morocco | 1111 | 1546 | 398 |
| Mauritius | 10 |  |  |
| Mauritania | 159 | 158 | 159 |
| Mexico | 64158 | 91617 | 63681 |
| Montenegro | 98 | 96 | 92 |
| Mozambique | 23 | 52 | 23 |
| ­Namibia | 72 | 216 | 72 |
| Nepal | 221 | 1043 | 198 |
| Nicaragua | 137 | 152 | 136 |
| Niger | 69 | 69 | 69 |
| Nigeria | 1013 | 1168 | 1034 |
| Norway | 264 | 251 | 251 |
| New Zealand | 22 | 22 | 22 |
| Oman | 677 | 919 | 675 |
| Uganda | 30 | 64 | 31 |
| Uzbekistan | 313 | 389 | 308 |
| Pakistan | 6288 | 6248 | 6228 |
| Panama | 1995 | 2008 | 1957 |
| Paraguay | 308 | 3960 | 314 |
| Netherlands | 6252 | 6151 | 6172 |
| Peru | 28607 | 112397 | 30017 |
| Philippines | 3520 | 19573 | 3191 |
| Poland | 2033 | 2051 | 1902 |
| Portugal | 1819 | 1742 | 1737 |
| Qatar | 197 | 202 | 196 |
| United Republic of Tanzania | 21 | 21 | 21 |
| Syrian Arab Republic | 109 | 1190 | 110 |
| Central African Republic | 61 | 59 | 60 |
| Republic of Moldova | 992 | 1375 | 980 |
| Democratic Republic of the Congo | 258 | 263 | 249 |
| Dominican Republic | 1681 | 7997 | 1689 |
| Czech Republic | 423 | 376 | 376 |
| Romania | 3578 | 14263 | 3424 |
| United Kingdom of Great Britain and Northern Ireland | 41586 | 41027 | 41157 |
| Rwanda | 16 | 73 | 15 |
| San Marino | 42 | 42 | 42 |
| Sao Tome and Principe | 15 | 14 | 14 |
| Senegal | 284 | 374 | 284 |
| Serbia | 711 | 720 | 715 |
| Sierra Leone | 70 | 68 | 68 |
| Singapore | 27 | 27 | 27 |
| Slovakia | 33 | 29 | 29 |
| Slovenia | 133 | 117 | 117 |
| Somalia | 98 | 94 | 94 |
| Sudan | 823 | 830 | 807 |
| South Sudan | 47 | 47 | 47 |
| Sri Lanka | 12 | 11 | 11 |
| Sweden | 5821 | 5827 | 5817 |
| Switzerland | 2005 | 1962 | 1969 |
| Suriname | 67 | 661 | 70 |
| Tajikistan | 68 | 59 | 59 |
| Chad | 77 | 75 | 75 |
| Thailand | 58 | 58 | 58 |
| Togo | 27 | 92 | 27 |
| Trinidad and Tobago | 21 | 9 | 9 |
| Tunisia | 76 | 53 | 53 |
| Turkey | 6326 | 5744 | 5732 |
| Ukraine | 2575 | 5162 | 2411 |
| Uruguay | 44 | 49 | 40 |
| Venezuela, Bolivarian Republic of | 381 | 548 | 381 |
| Viet Nam | 32 | 32 | 30 |
| West Bank and Gaza | 152 | 165 | 137 |
| Yemen | 564 | 587 | 559 |
| Zambia | 287 | 311 | 292 |
| Zimbabwe | 196 | 249 | 192 |
