# Supplementary figures and images for: Covid-19 Mortality: A Matter of Vulnerability Among Nations Facing Limited Margins of Adaptation
Source: Front Public Health. 2020 Nov 19;8:604339. doi: 10.3389/fpubh.2020.604339 (PMC7710830; doi:10.3389/fpubh.2020.604339)

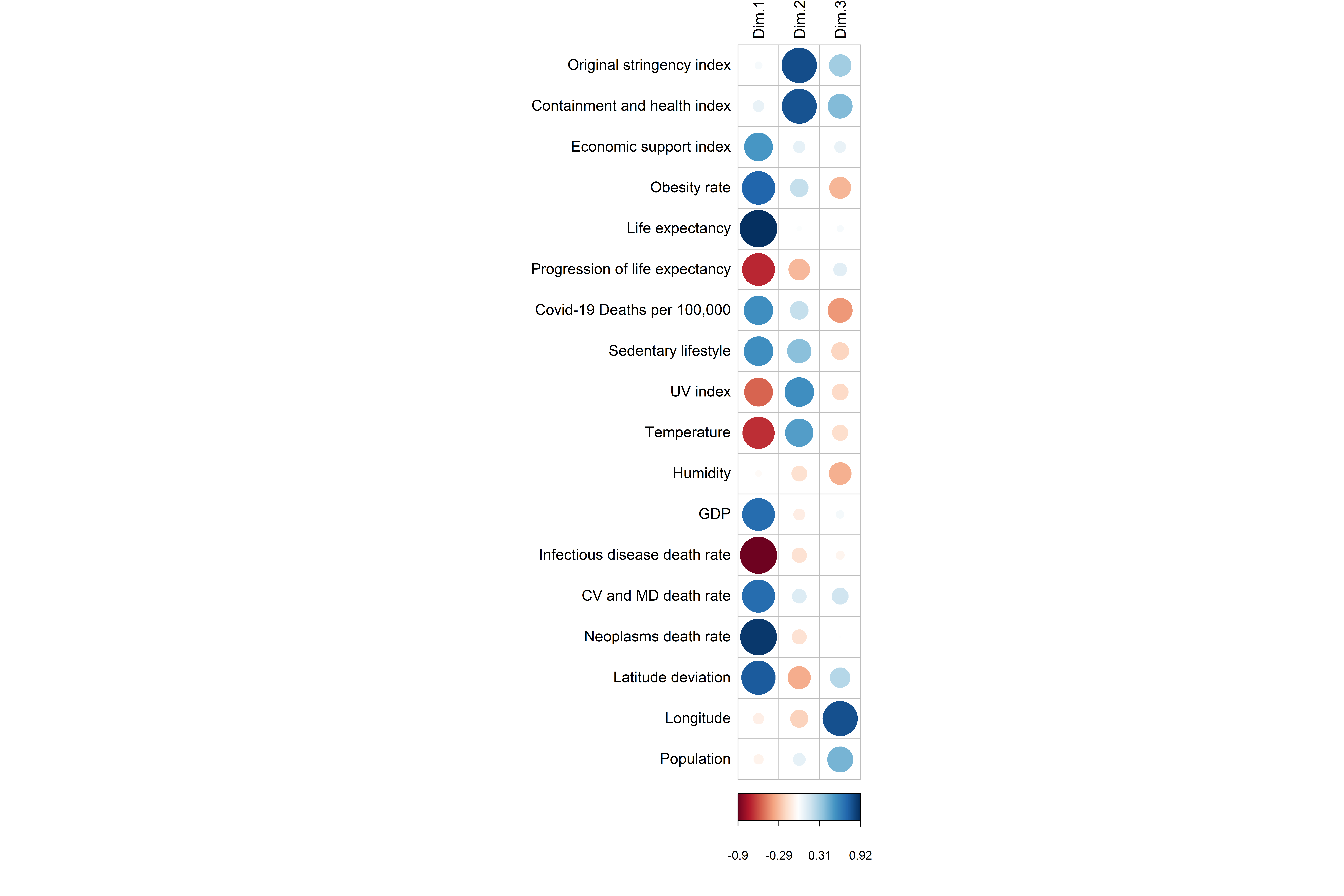

Supplement: Supplementary Figure 2 — Coordinates of the variables of the principal component analysis with the estimated data (see Section Methods—Data collection). Results are the same with estimated data and actual data. [file Image_2.TIFF]

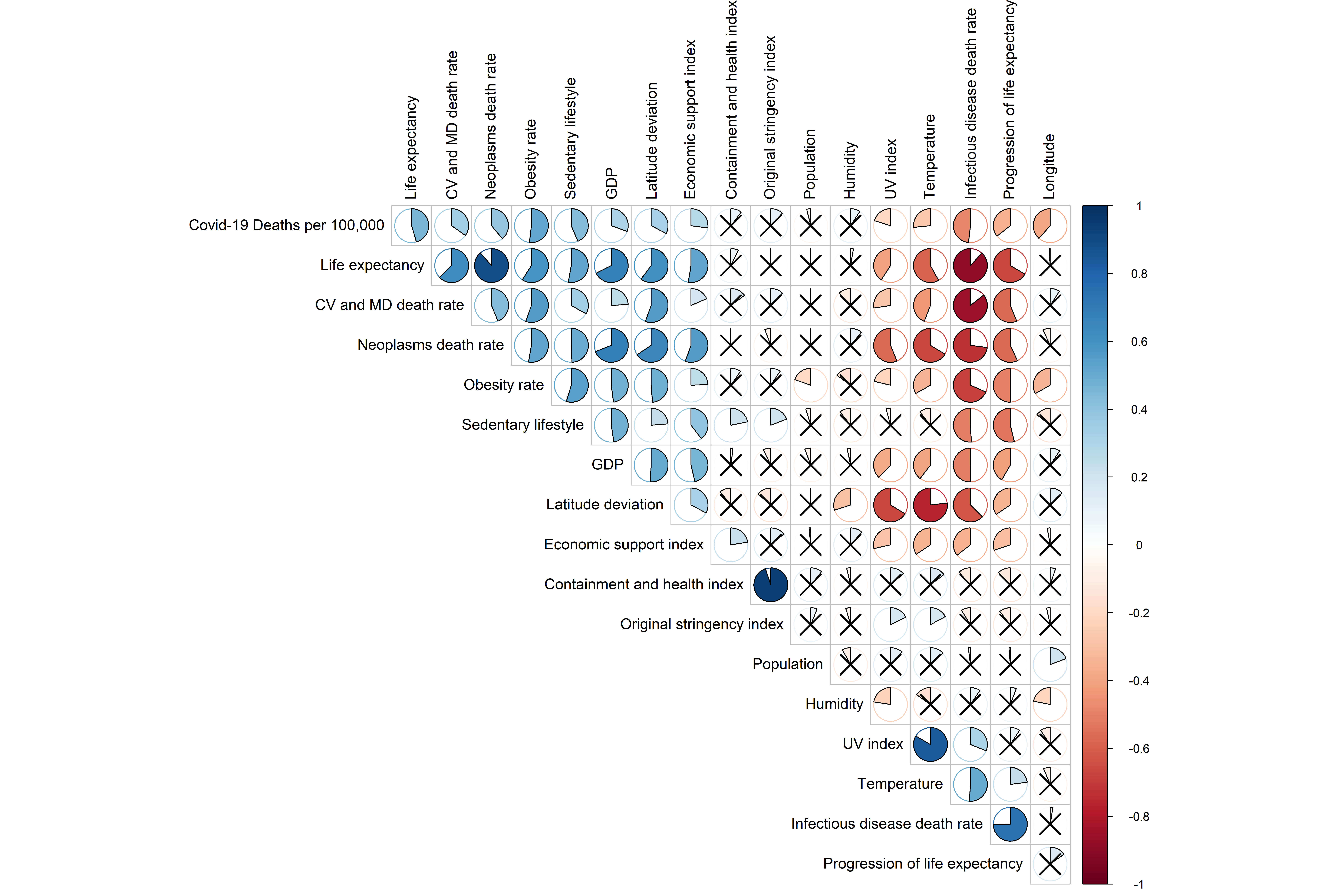

Supplement: Supplementary Figure 3 — Correlation matrix with the estimated death numbers obtained from the logistic equation (see Section Methods—Data collection). Results are the same with estimated data and actual data. [file Image_3.TIFF]

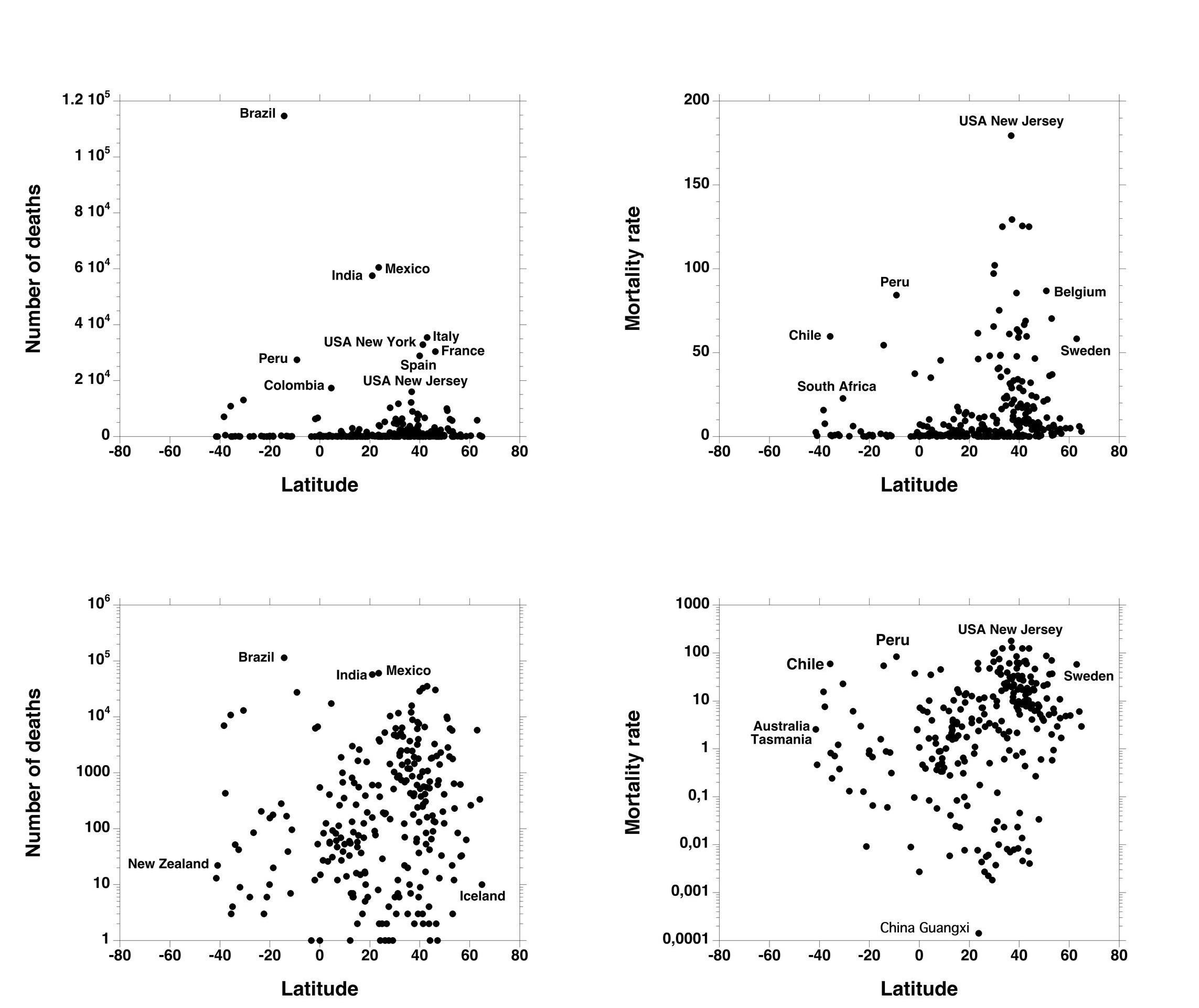

Supplement: Supplementary Figure 4 — Covid-19 mortality is expressed in numbers in the top figures and in rates in the bottom figures. It is expressed in raw data on the left and in decimal logarithm on the right. [file Image_4.JPEG]

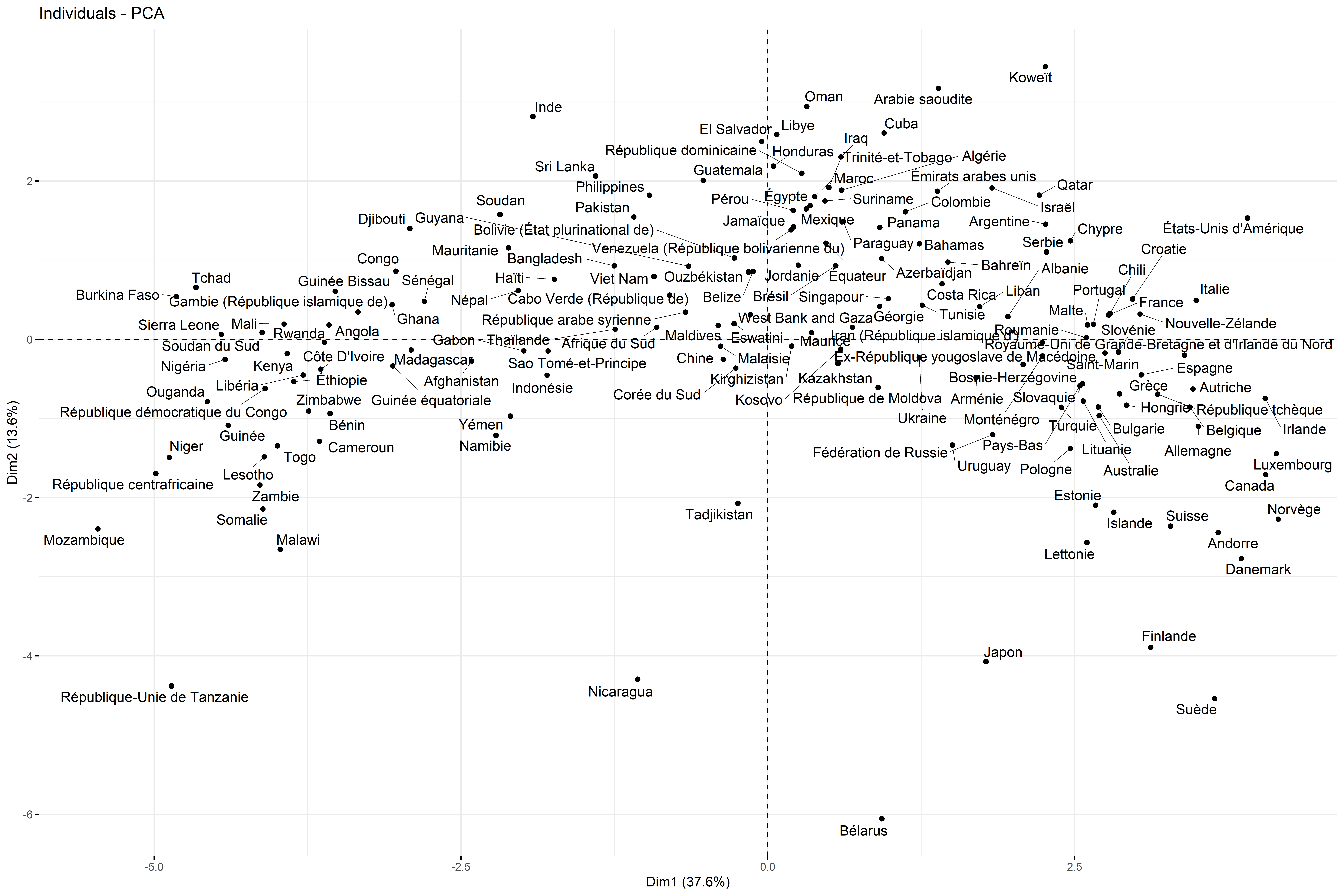

Supplement: Supplementary Figure 5 — The position of the countries on the graph represents their correlation according to the variables of the first factorial plane. For example, countries positively correlated to axis 1 (right), will be positively correlated to variables to the right of axis 1. [file Image_5.TIFF]
